# MicroRNAs as regulators of cisplatin-resistance in non-small cell lung carcinomas

**DOI:** 10.18632/oncotarget.22975

**Published:** 2017-12-05

**Authors:** Irina Fadejeva, Horst Olschewski, Andelko Hrzenjak

**Affiliations:** ^1^ Division of Pulmonology, Department of Internal Medicine, Medical University of Graz, Graz, Austria; ^2^ Ludwig Boltzmann Institute of Lung Vascular Research, Medical University of Graz, Graz, Austria

**Keywords:** cisplatin resistance, microRNA, chemotherapy, NSCLC, hypoxia

## Abstract

With more than 80% of all diagnosed lung cancer cases, non-small cell lung cancer (NSCLC) remains the leading cause of cancer death worldwide. Exact diagnosis is mostly very late and advanced-stage NSCLCs are inoperable at admission. Tailored therapies with tyrosine kinase inhibitors are only available for a minority of patients. Thus, chemotherapy is often the treatment of choice. As first-line chemotherapy for NSCLCs, platinum-based substances (e.g. cisplatin, CDDP) are mainly used. Unfortunately, the positive effects of CDDP are frequently diminished due to development of drug resistance and negative influence of microenvironmental factors like hypoxia. MicroRNAs (miRNAs) are small, non-coding molecules involved in the regulation of gene expression and modification of biological processes like cell proliferation, apoptosis and cell response to chemotherapeutics. Expression of miRNAs is often deregulated in lung cancer compared to corresponding non-malignant tissue. In this review we summarize the present knowledge about the effects of miRNAs on CDDP-resistance in NSCLCs. Further, we focus on miRNAs deregulated by hypoxia, which is an important factor in the development of CDDP-resistance in NSCLCs. This review will contribute to the general understanding of miRNA-regulated biological processes in NSCLC, with special focus on the role of miRNA in CDDP-resistance.

## INTRODUCTION

Lung cancer is a very common cancer worldwide [1]. There are different types of lung cancer, that are diagnosed by histology and cytology [2]. The final diagnosis should be determined multidisciplinary by analyzing morphology and genetic changes of the cancerous tissue. Classification of lung cancer includes two large histological groups: non-small cell lung cancer (NSCLC) accounting for approx. 85%, and small cell lung cancer (SCLC) accounting for approx. 15% of all lung cancers [3]. NSCLC are classified into three different subtypes: adenocarcinoma (ADC), squamous-cell carcinoma (SCC) and large-cell carcinoma (LCC) [3–5]. Adenocarcinoma is the most frequent type of lung cancer [6]. Mortality is very high, with 5-year survival rates around 15–20%. This is due to both poor tools for early diagnosis where the disease is still operable, and poor tools for therapy, once the tumor is inoperable. The therapy for NSCLC patients frequently includes platinumbased chemotherapeutics like cisplatin (cis-diaminedichloroplatinum, CDDP) or carboplatin. It is well known that continuous and/or multiple administrations frequently result in the development of drug resistance, which often leads to treatment failure.

Accumulating data indicate that CDDP-resistance is *inter alia* modified by microRNAs (miRNAs). MiRNAs are small, endogenous, noncoding RNA molecules that consist of about 18–23 nucleotides and have influence on posttranscriptional regulation of gene expression, thereby acting as tumor suppressor or as oncogenes [7]. Evolutionary conserved, miRNAs bind to the 3´-untranslated region (3´-UTR) of target mRNA, leading to translational repression and mRNA degradation. MiRNAs play a vital role in different cellular processes in non-malignant and in tumor cells, such as cell growth, differentiation, motility and apoptosis. MiRNAs in cancer are involved in different processes of tumorigenesis like tumor proliferation, migration, angiogenesis, apoptosis, drug transport, DNA repair, etc. [8]. MiRNAs are involved in the development of a variety of tumors, such as leukemia, neuroblastoma, pituitary adenoma, breast cancer, thyroid cancer, hepatocarcinoma, colorectal cancer, and lung cancer. The up- or down-regulation of miRNAs in different tumor tissues has been shown, with most of the miRNA targets located in regions of tumor-related genes, fragile sites, loss of heterozygosity, and amplified regions. For example miR-21 is overexpressed in many human malignancies, including NSCLC [9].

The molecular and genetic basis of sensitivity and resistance to chemotherapy is complex, involving multiple processes such as regulation of cell cycle, apoptosis, drug transport, drug metabolism, DNA repair, etc. The molecular mechanisms of CDDP-resistance have not been fully understood and may include: decreased accumulation of CDDP, increased detoxification systems (such as GSH, GSTP1, and metallothionein), decreased DNA damage, and/or increased DNA repair. CDDP-resistance in tumor cells allows the cells to escape the cytotoxic effects of the drug and to overcome apoptosis [10]. In lung cancer, it has been shown that miRNAs play an important role in the development of chemosensitivity and chemoresistance [11]. In tumor cells and tumor tissues these regulatory mechanism are complementary and can either enhance or block each other. This review article will describe the role of miRNAs in CDDP-resistance of NSCLC cells.

### MiRNAs and cell proliferation in CDDP-resistant NSCLCs

**Figure 1:**
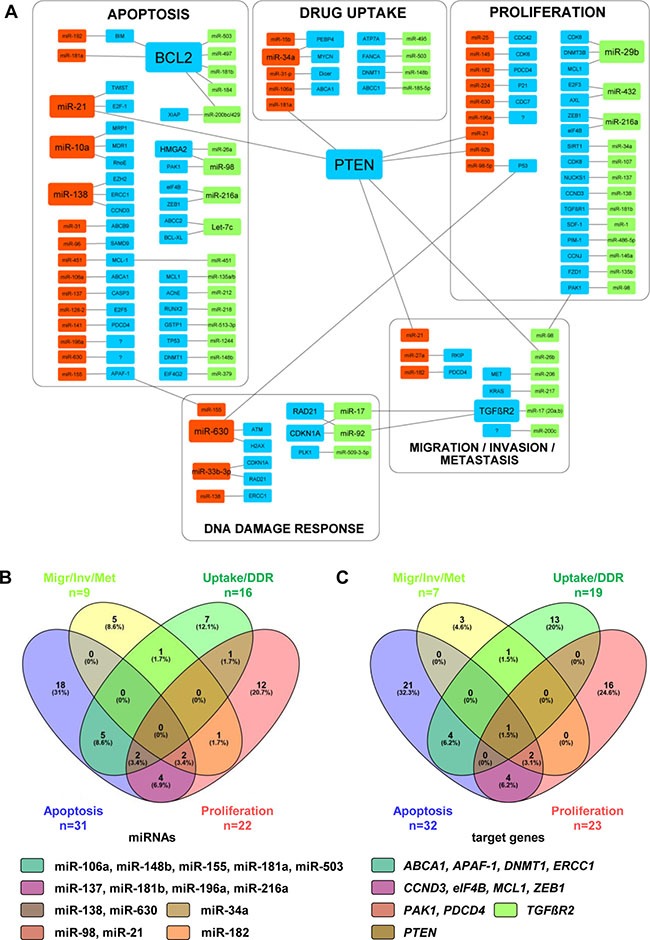
Correlations between miRNAs involved in resistance of NSCLC cells to CDDP (**A**) Different miRNAs and their target genes listed in Tables 1-3 were graphically put in correlation by using the Cytoscape software (ver. 3.4.0). Size of rectangular shapes correlates to the number of interactions between miRNAs and target genes. Red, up-regulated miRNAs; green, down-regulated miRNAs; blue, target genes. (B, C) Venn diagrams showing correlations between the miRNAs (*n* = 78) within particular pathways (**B**) and between target genes (*n* = 81) assigned to particular pathways (**C**). All MiRNAs and target genes shown in Venn diagrams are named in particular subgroups shown in Figure 1A. Venn diagrams were generated by publically available Venny-tool (http://bioinfogp.cnb.csic.es/tools/venny/). The group Apoptosis is shown in violet, group Proliferation in pink, group Migration/Invasion/Metastasis (Migr/Inv/Met) in yellow, and group Drug uptake/DNA repair (Uptake/DDR) in green. For each particular subgroup, the number and percentage of miRNAs or target genes are indicated. MiRNAs and target genes detected as multiplayers in different cell processes are listed accordingly to the color code of Venn diagrams.

One single miRNA can regulate different target genes, and one target gene can be regulated by different miRNAs, making the assignment of one miRNA to a particular pathway or to a molecular mechanism very challenging. This is especially the case for miRNAs and their target molecules involved in cell proliferation and apoptosis, mechanisms of extraordinary importance for tumor development and progression. Figure 1 summarizes correlations between different miRNAs and their target genes known to be involved in resistance of NSCLC cells to CDDP. It clearly indicates that many miRNAs influence different target genes and are, therefore, players in different cellular processes. In context of the CDDP-resistance in NSCLC cells, miR-21 appears as very prominent. MiR-21 influences target genes involved in apoptotic pathways, cell proliferation, migration, invasion, and metastasis development. Among target genes regulated by different miRNAs, PTEN is particularly prominent, and appears to be involved in the regulation of CDDP-resistance in NSCLC cells and tumors. These regulatory mechanisms and their possible correlations will be discussed in more detail in the following paragraphs.

Tumor progression is dependent on different cellular processes, including cell proliferation and apoptosis. MiRNA-216a regulates NSCLC progression by acting as a tumor suppressor. Wang et al. analyzed 119 NSCLC specimens and detected significant down-regulation of miR-216a compared to adjacent lung tissue [12]. Furthermore, this down-regulation was associated with the tumor stage and the development of metastasis in NSCLC patients. By means of different experimental approaches it was shown that miR-216a suppresses cell growth by affecting cell proliferation and apoptosis, both *in vitro* and *in vivo*. Primary tumors from NSCLC patients with metastasis showed significantly decreased miR-216a expression in comparison to patients without metastasis. As main target-genes eIF4B and ZEB1, two oncogenes playing an important role in chemoresistance, were identified. This was further supported by marked increase of the sensitivity of NSCLC cells to CDDP treatment due to miR-216a overexpression. Surprisingly, in liver cancer not eIF4B or ZEB1, but PTEN and SMAD7 were the main targets of miR-216a. These discrepancies might be based on different algorithms used for target gene prediction, or more probably, they indicate that the same miRNA targets different genes in different tissues.

MiR-630 inhibits activation of p53, the master regulator of CDDP-induced cell death, and blocks the early DNA damage response in lung cancer cells [13]. Furthermore, miRNA-630 also reduces pro-apoptotic pathways regulated by p53. Pre-miR-630–transfected cells were arrested in the G0-G1 phase, which correlated with increased levels of the p27Kip1 protein. P27Kip1 is a cell cycle inhibitor that regulates cell cycle progression at the G1 phase due to interaction with cyclin-CDK2 and -CDK4. Interestingly, not only the proliferation rate, but also the sensitivity of cells overexpressing pre-miR-630 to CDDP was markedly reduced. This suggests that miR-630 protects NSCLC cells against CDDP by regulating the same signaling pathway as p53.

MiR-34a was significantly up-regulated in CDDP-treated NSCLC tumor tissues and NSCLC cell lines, indicating that CDDP induces miR-34a expression. Expression of miR-34a is regulated by p53 and miR-34a regulates apoptosis, cell cycle and differentiation [14, 15]. MiR-34a expression was significantly down-regulated in NSCLC tumor tissue in comparison to adjacent non-malignant lung tissue. Interestingly, miR-34a level in serum of CDDP-sensitive patients was higher in comparison to patients with CDDP-resistance. The MYCN gene, coding for N-myc proto-oncogene protein is a direct functional target of miR-34a. Altogether, these data indicate that miR-34a enhances the sensitivity of NSCLC cells to CDDP via the p53/miR-34a/MYCN axis.

Different studies suggested an up-regulation of miR-92b in NSCLC tissue and cell lines, compared to adjacent lung tissue. Overexpression of miR-92b promoted A549/CDDP cell growth and inhibited apoptosis, thereby inducing CDDP-resistance by directly targeting the tumor suppressor gene PTEN [16]. Inhibition of miR-92b had the opposite effects. In another study, almost identical effects have been described, however, the expression of miR-92b in NSCLC tissues was negatively correlated with the tumor suppressor gene RECK which is frequently down-regulated in tumors [17]. Together, all these results suggest that miR-92b acts as an oncogene in the NSCLC.

Overexpression of polo-like kinase 1 (PLK1) is a negative prognostic factor for NSCLC patients and patients suffering from some other solid tumors, like esophageal carcinoma, head and neck tumors and melanoma [18]. PLK1 is known as a DNA-damage checkpoint modulator that maintains genomic stability. PLK1 expression is correlated to cancer aggressiveness, based on fast growth of cancer cells [19]. In human lung cancer A549 cells miR-509-3-5p downregulates PLK1 expression by targeting PLK1 3´-UTR and acting as a cancer suppressor [20]. Moreover, PLK1 downregulation upon overexpression of miR-509-3-5p in A549 cells results in G2/M arrest and aberrant mitosis rate [20].

Uncontrolled cell division is a typical feature of cancer cells. Cell cycle is regulated by different proteins and members of the cyclin-dependent protein kinase (CDK) family, known to be relevant regulatory factors in cell cycle progression. One of these proteins, CDK8, is a target of miR-107 in NSCLC cells. Down-regulation of CDK8 in NSCLC cell lines is associated with enhanced sensitivity to CDDP. This suggests that miR-107 takes part in chemotherapy resistance by negative regulation of CDK8, and might be used to predict a NSCLC patient’s response to chemotherapy [21]. Another member of the CDK family, CDK6, is a target of miR-145 and downregulation of CDK6 is positively correlated with CDDP-resistance in NSCLC cells [22]. These findings might be of great importance, indicating that agents which suppress CDK6 should not be used during CDDP therapy.

It was shown that CDC7 (cell division cycle 7-related protein kinase), a protein involved in the cell cycle regulation, acts as a target of miR-630, which binds to four binding sites in CDC7 3´-UTR. MiR-630 inhibits the expression of CDC7, thereby blocking the initiation of DNA synthesis, inducing the G1 arrest, and promoting apoptosis. Interestingly, miR-630 can also reduce apoptosis by blocking other apoptotic modulators such as DDIT4, PARP3 and EP300. Altogether, these findings suggest the bimodal role of miR-630 in the regulation of apoptosis and CDCP-mediated resistance in NSCLC cells [23]. CDC42 is a target of miR-25, taking part in cell proliferation and CDDP-resistance development. Down-regulation of miR-25 inhibits proliferation of NSCLC cells, promotes G1 arrest and enhances CDDP-sensitivity via downregulation of CDC42 *in vitro*. *In vivo* experiments showed that downregulation of miR-25 reduces tumor growth rate [24].

MiRNAs involved in CDDP-resistance of NSCLC cells which are mainly assigned to cell proliferation and cell cycle pathways are summarized in Table 1.

**Table 1:** MiRNAs involved in CDDP-resistance of NSCLC cells mainly assigned to cell proliferation and cell cycle pathways

miRNA	Targets	Cells / Tissue	Expression	Function	Reference
miR-1	SDF-1	Cancer-associated fibroblasts (CAFs)	down	Negatively regulates SDF-1 and influence CDDP-resistance in CAFs	[106]
mR-21	PTEN	Serum, NSCLC cells	up	Correlates with TNM stage, increases growth	[9]
miR-25	CDC42	NSCLC tissue, A549/PAR	up	Increases proliferation, decreases CDDP-sensitivity	[24]
miR-29b	CDK6, DNMT3B, MCL1	A549 cells	down	Reduces expression of CDK6, inhibits cell growth	[107]
miR-34a	SIRT1	NSCLC tissue, A549/PAR	down	Sensitizes NSCLC cells to CDDP	[108]
miR-92b	PTEN	NSCLC tissue, A549/PAR	up	Oncogene, inhibits apoptosis, induces cell growth and resistance to CDDP	(46)
miR-98	PAK1	NSCLC tissue, A549/PAR	down	Inversely regulates PAK1, inhibits proliferation	(87)
miR-98-5p	P53	NSCLC cells	up	Inhibition of miR-98-5p increases p53 expression and CDDP efficacy	[109]
miR-107	CDK8	A549/PAR	down	Neg. regulates CDK8, inhibits cell growth, has a key role in CDDP-resistance	[21]
miR-135b	FZD1	A549/CDDP	down	Suppresses CDDP-resistance in NSCLC by targeting FZD1	[110]
miR-137	NUCKS1	NSCLC tissue, A549/CDDP	down	Down-regulation promotes cell growth, migration and G1/S transition	[111]
miR-138	CCND3	NSCLC cells	down	Negatively regulates CDDP-resistance, influences cell migration	[40]
miR-145	CDK6	NSCLC cells/CDDP	up	Indirectly influences DCK6 expression and CDDP-resistance	[22]
miR-146a	CCNJ	A549/CDDP	down	Increases the sensitivity to CDDP, targets drug-resistance-associated proteins	[112, 113]
miR-181b	TGFßR1	A549/CDDP	down	Influence CDDP-resistance by targeting TGFßR1/Smad pathway	[114]
miR-182	PDCD4	A549/PAR	up	Inversely regulates PDCD4 expression, influences CDDP-resistance	[84]
miR-196a	n. d.	A549/CDDP	up	Regulates MDR1, MRP1, ERCC1, surviving, Bcl2 and RhoE	[115]
miR-216a	eIF4B, ZEB1	NSCLC tissue	down	Tumor suppressor miRNA, influences CDDP-induced apoptosis	[12]
miR-224	P21(WAF1/CIP1)	A549/CDDP	up	Promotes CDDP-resistance via regulating G1/S transition and apoptosis	[116]
miR-432	E2F3, AXL	NSCLC tissue, NSCLC cells	down	Negatively correlates with E2F3 and AXL and influence CDDP-resistance	[117]
miR-486-5p	PIM-1	NSCLC cells, NSCLC tissue	down	Negatively regulates Pim-1 and decreases sensitivity to CDDP	[118]
miR-630	CDC7	NSCLC cells	up	Has a bimodal role in regulation of DNA-damage and apoptosis	[23]

n. d., not defined; Expression = expression in CDDP-resistant cells or NSCLC tissue in comparison to sensitive cells or non-malignant lung tissue.

### MiRNAs and apoptosis in CDDP-resistant NSCLCs

MiRNAs can influence different cell events like differentiation, proliferation, metabolism, growth, and cell death [25]. Mechanisms of the DNA damage and cell apoptosis are closely connected. DNA damage activates the stimulation of checkpoint mechanisms and leads to DNA repair or to cell death by apoptosis [26]. Different studies suggested that miRNAs control cell cycle by activating or inhibiting different proteins involved in a DNA damage response. Members of the BCL-2 family are between the most frequently described target molecules of different miRNAs concerning apoptosis-related CDDP-resistance in NSCLC cells (for summary see Fig. 1A). MiRNAs involved in CDDP-resistance of NSCLC cells, which are mainly assigned to apoptosis pathways, are summarized in Table 2. Many of these miRNAs have different target genes. However, for more clarity, only target genes proven to be involved in CDDP-resistance in NSCLCs are listed here.

**Table 2:** MiRNAs involved in CDDP-resistance of NSCLC cells mainly assigned to apoptosis pathway

miRNA	Targets	Cells / Tissue	Expression	Function	Reference
miR-10a	RhoE, MDR1, MRP1	A549/CDDP	up	Inhibits apoptosis, enhances drug efflux	[62]
miR-21	E2F-1, TWIST, PTEN	NSCLC tissue, NSCLC cells, A549/CDDP	up	Inhibits apoptosis, blocks the cell cycle, increases CDDP-resistance	[54, 119, 120]
miR-26a	HMGA2	A549/CDDP	down	Regulates E2F1-Akt pathway	[121]
miR-31	ABCB9	CDDP-resistant NSCLC cells	up	Anti-apoptotic effects via ABCB9 inhibition	[67]
miR-96	SAMD9	NSCLC tissue, NSCLC cells	up	Down-regulates SAMD9 expression, decreases apoptosis	[64]
miR-98	HMGA2, PAK1	A549/CDDP, NSCLC tissue, A549/PAR	down	Modulates apoptosis via HMGA2, inversely regulates PAK1, inhibits apoptosis	[50, 51]
miR-106a	ABCA1	A549/CDDP	up	Represses apoptosis, decreases CDDP-uptake	[68]
miR-128-2	E2F5	NSCLC cells	up	Inhibits apoptosis, increases resistance to CDDP	[61]
miR-135a/b	MCL1	A549/CDDP	down	Modulates apoptosis via MCL1, overexpression sensitizes A549/CDDP	[49]
miR-137	CASP3	NSCLC cells overexpressing miR-137	up	Decreases CASP3 expression and cell death	[122]
miR-138	ERCC1, EZH2, CCND3	A549/CDDP	up	Increases sensitivity to CDDP and apoptosis	[38–40]
miR-141	PDCD4	A549/CDDP vs. A549/PAR	up	Contributes to CDDP-resistance by suppressing PDCD4	[65]
miR-148b	DNMT1	A549/CDDP, SPC-A1/CDDP	down	Influence CDDP-sensitivity, cell viability and apoptosis	[57]
miR-155	APAF-1	NSCLC tissue, A549/PAR	up	Inhibits sensitivity to CDDP via negative regulation of Apaf-1	[37]
miR-181a	n. d.	CDDP- treated A549/PAR	up	Enhances CDDP-triggered mitochondrial apoptosis	[13]
miR-181b	BCL2	A549/CDDP	down	Modulation of CDDP-induced apoptosis via targeting BCL2	[123]
miR-184	BCL2	NSCLC tissue	down	Decrease of miR-184 by E6 predicts unfavorable response to CDDP	[30]
miR-192	BIM, BCL2	A549/PAR, A549/CDDP	up	Inhibits apoptosis via Bcl2, induces CDDP-resistance	[28, 29]
miR-196a	n. d.	NSCLC tissue, NSCLC cells	up	Increases CDDP-resistance by inhibiting apoptosis	[124]
miR-200bc/429 cl.	BCL2, XIAP	A549/CDDP	down	Inversely regulates BCL2/XIAP expression and apoptosis	[125]
miR-212	AChE	CDDP-treated A549/PAR	down	Anti-apoptotic effects through AChE repression	[60]
miR-216a	eIF4B, ZEB1	NSCLC tissue	down	Suppresses NSCLC growth and metastasis, enhances apoptosis	[12]
miR-218	RUNX2	NSCLC tissue, NSCLC cells	down	Increases resistance to CDDP via targeting RUNX2	[63]
miR-379	EIF4G	NSCLC tissue, NSCLC cells	down	Tumor suppressor, influences CDDP-induced apoptosis by targeting EIF4G	[66]
miR-451	MCL-1	NSCLC tissue, A549/PAR, A549/CDDP	up / down	Tumor suppressor, inhibits growth and apoptosis by inactivating Akt pathway	[47, 48]
miR-497	BCL2	A549/CDDP	down	Modulation of CDDP-induced apoptosis via targeting BCL2	[35]
miR-503	BCL2	A549/CDDP	down	Increases resistance to CDDP via targeting BCL2	[55]
miR-513-3p	GSTP1	A549/CDDP	down	Enhances CDDP-induced apoptosis	[126]
miR-630	n. d.	CDDP- treated A549/PAR	up	Reduces CDDP-triggered mitochondrial apoptosis	[13]
miR-1244	TP53	A549/CDDP, A549/PAR	down	Reduces cell proliferation, survival and invasion	[83]
Let-7c	ABCC2, BCL-XL	A549/CDDP	down	Modulates CDDP-response by targeting ABCC2 and BCL-XL	[36]

cl., cluster; n. d., not defined; Expression = expression in CDDP-resistant cells or NSCLC tissue in comparison to sensitive cells or non-malignant lung tissue.

Up-regulation of Bim, an essential component of the electron transport chain, leads to cytochrome-c release from mitochondria and, consequently, to activation of the mitochondrial apoptosis pathway. Accordingly, post-translational down-regulation of Bim by miR-192 leads to inhibition of apoptosis and to CDDP-resistance in lung adenocarcinoma cells [27, 28]. Furthermore, miR-192 directly targets Bcl-2, thereby influencing apoptosis and resistance of A549 cells in response to cisplatin and gemcitabine, a therapy combination frequently used for NSCLC [29]. Down-regulation of miR-184, which is regulated by E6 oncoprotein, can also be involved in the development of CDDP resistance in NSCLC cells. This supposes that down-regulation of miR-184 may play a role in CDDP resistance by increasing the Bcl-2 expression and thus influencing apoptosis [30]. In NSCLC tissues and cell lines, miR-497 targets different genes like vascular endothelial growth factor A (VEGF-A) [31], yes-associated protein 1 (YAP1) [32], cyclin E1 (CCNE1) [33], hepatoma-derived growth factor (HDGF) [34], and Bcl-2 [35]. However, only Bcl-2 repression by miR-497 was involved in the modulation of CDDP-resistance in NSCLC cells [35].

Bcl-XL is an antiapoptotic protein, shown to be directly deregulated by let-7c. The role of let-7c in development of CDDP-resistance and -sensitivity in NSCLC was investigated by Zhan et al. They showed that let-7c modulates the CDDP-response by targeting ABCC2 and Bcl-XL. The sensitivity of A549/CDDP cells to CDDP was increased upon down-regulation of let-7c [36]. In lung cancer tissue, significantly higher miR-155 expression was detected compared to adjacent non-malignant lung tissue [37]. Apaf-1, a protein that stimulates caspase-9 and activates apoptosis, also can act as a target of miR-155, and up-regulation of miR-155 inhibits the sensitivity of NSCLC cells to CDDP by modulating the DNA damage response (DDR) and apoptosis. After CDDP treatment of miR-155-silenced A549 cells the Apaf-1 protein, but not the Apaf-1 mRNA, was highly elevated. Both the miR-155-silencing and Apaf-1 overexpression markedly increased the sensitivity of A549 cells to CDDP, and this was correlated with increased apoptosis and DNA damage. MiR-155 in NSCLC tissue compared to paracancerous and non-malignant lung tissue expression is significantly increased [37]. Down-regulation of miR-155 increased the sensitivity to CDDP by targeting apoptotic protease activating factor 1 (Apaf-1). Down-regulated miR-155 correlated with high levels of the Apaf-1, an activator of cell apoptosis. This suggests that miR-155 might play a role in the NSCLC carcinogenesis and influence chemoresistance by induction of apoptosis and DDR.

Many studies have examined the expression of miR-138 in NSCLC and its influence on CDDP-resistance. Upregulated miR-138 increased the sensitivity to CDDP by inducing apoptosis [38]. In A549/CDDP cells there was a negative correlation between miR-138 and a DNA excision repair cross-complementation group protein (ERCC1). ERCC1 is a part of the enzyme complex ERCC1-XPF responsible for the DNA repair. Consequently, down-regulation of ERCC1 by overexpressed miR-138 influenced the CDDP-resistance in A549/CDDP cells. This agrees to the down-regulation of miR-138 in NSCLC tissue and four different NSCLC cell lines in comparison to non-malignant lung tissue [39]. Moreover, overexpression of miR-138 in NSCLC cell suppresses cell proliferation and induces apoptosis *in vitro* and suppresses NSCLC tumor growth in a xenograft mouse model, indicating miR-138 as a potential tumor suppressor. Interestingly, in this study the enhancer of zeste homolog 2 (EZH2) was identified as a direct target of miR-138. The EZH2, an oncogene with a histone-lysine N-methyltransferase activity, has been linked to initialization and propagation of different malignancies. Upregulated miR-138 can inhibit cell mitosis and cell growth in NSCLC and increase sensitivity to CDDP by directly regulating cyclin D3 (CCND3) [40]. Further genes identified as targets of miR-138 in NSCLC tissues and cell lines are: 3-phosphoinositide-dependent protein kinase-1 (PDK1) [41], forkhead box P4 (FOXP4) [42], G-protein-coupled receptor kinase-interacting protein 1 (GIT1) and semaphoring 4C (SEMA4C) [43], yes-associated protein 1 (YAP1) [44], and LIM domain kinase (LIMK1) [45]. However, it is not known whether these target genes play any role in CDDP-resistance of NSCLC cells.

Galluzzi et al. showed that miR-181a increases sensitivity of A549 cells to CDDP by stimulating Bax oligomerization and activation of caspases, thus activating mitochondrial apoptosis. The same study showed that Mir-630 has a protective role against CDDP, due to partial inhibition of p53-regulated pro-apoptotic signaling pathways in A549 cells [13]. Recently, it has been shown that overexpressed miR-181a promotes invasion and migration of lung adenocarcinoma cells by directly targeting PTEN. It influences metastatic features, epithelial-mesenchymal transition (EMT) and promotes CDDP-resistance in lung adenocarcinoma cells [46]. These findings suggest miR-181a as an interesting target for development of new therapeutic strategies in NSCLC patients, both alone, as well as in combination with existing chemotherapies.

MiR-451 targets many different genes involved in cell proliferation, apoptosis, EMT and chemoresistance of lung adenocarcinoma. The level of miR-451 was reduced in CDDP-resistant in comparison to parental lung carcinoma cell lines. Bian et al. showed in their *in vitro* and *in vivo* experiments that upregulation of miR-451 inhibits growth of NSCLC cells and increases their sensitivity to CDDP by inducing cell apoptosis [47]. Furthermore, overexpression of miR-451 sensitizes A549/CDDP cells to CDDP by directly targeting MCL-1, an anti-apoptotic protein and member of the Bcl-2 family, and suppresses A549 cells derived xenograft tumor growth in the presence of CDDP [48]. Altogether, this suggests that miR-451 has a tumor suppressive role in NSCLC cells. Bcl-1 is also a target of miR-135a/b, which is down-regulated in CDDP-resistant A549 cells [49]. In A549 cells transfected with miR-135a/b mimics, the MCL1 expression was decreased, and so was the cell resistance to CDDP. These data indicate that miR-135a/b can at least partially sensitize CDDP-resistant NSCLC cells to CDDP by repressing MCL1 and influencing apoptosis.

Several studies found dysregulation of miR-98, which also modulates CDDP-resistance. Expression of miR-98 induces apoptosis by targeting high mobility group A2 (HMGA2) oncogene, thus making cells more sensitive to CDDP [50]. Another study demonstrated that miR-98 was down-regulated in NSCLC tissue (*n* = 8) compared to adjacent non-malignant lung tissue. Mir-98 inhibits proliferation, cell migration and invasion, and induces apoptosis in human NSCLC cells. MiR-98 negatively regulates P21-activated protein kinase (PAK1) at the posttranslational level, and therefore inhibits cell growth and proliferation in NSCLC cells [51].

Based on elevated expression level in plasma samples of NSCLC patients, MiR-21 was suggested as a potential biomarker for diagnosis of NSCLC at an early stage, and as a predictive indicator for the sensitivity to platinum-based chemotherapy [52]. In numerous studies elevated miR-21 expression in NSCLC cell lines and tissue specimens was shown [9, 53, 54]. Elevated miR-21 expression is related to increased growth, migration and invasion, whereas its down-regulation inhibits these processes in NSCLC cells. It has been found that miR-21 promotes CDDP-resistance by inhibiting apoptosis via directly targeting PTEN, the expression of which is frequently downregulated in NSCLC tissues [9, 54]. As shown in Fig. 1A, PTEN is one of the most prominent target genes involved in resistance of NSCLC cells to CDDP, whose expression is regulated by many different miRNAs. In addition, it has been shown that NF-κB regulates the expression of miR-21 by binding its promoter, strongly indicating the importance of NF-κB/miR-21/PTEN pathway in development of CDDP-resistance in NSCLC cells [54]. Thus, down-regulation of miR-21 might provide a new potential strategy for treatment of NSCLC patients.

Resistance of non-small cell lung cancer cells to CDDP is also mediated by miR-503. The expression of miR-503 is decreased in the CDDP-resistant non-small cell lung cancer cells compared with the parental cells. The over-expression of miR-503 sensitizes the A549/CDDP cells to CDDP, whereas the inhibition of miR-503 in the A549 cells increases resistance to CDDP. MiR-503 specifically targets Bcl-2, an anti-apoptotic protein upregulated in A549/CDDP cells. The ectopic expression of miR-503 reduces the Bcl-2 protein level and sensitizes the A549/CDDP cells to CDDP-induced apoptosis [55]. Another study showed that miR-503 regulates the resistance of non-small cell lung cancer cells to CDDP, at least in part, by targeting FANCA (Fanconi anemia complementation group A protein). This suggests that targeting FANCA may provide a novel strategy for the sensitization of NSCLC to CDDP [56]. Lung cancer cells may express increased levels of FANCA. Overexpression of FANCA was indirectly associated with the hypermethylation and downregulation of miR-503 in NSCLC. In CDDP-resistant cells the level of DNA (cytosine-5)-methyltransferase 1 (DNMT1) was increased, suggesting that enhanced expression of DNMT1 takes part in methylation of miR-503.

Mir-148b, a member of miR-148/152 family, was found to be notably down-regulated in CDDP-resistant NSCLC cells [57]. DNMT1 was detected as a direct target of miR-148b. DNMT1 mediates the transfer of methyl groups from S-adenosylmethionine to the 5 position of cytosine bases in the dinucleotide sequence CpG [58], thus mediating transcriptional silencing in cancer cells. MiR-148b exerts negative effects on DNMT1 expression by targeting its 3′-UTR in A549/CDDP cells. Furthermore, silencing DNMT1 increased the sensitivity of A549/CDDP cells to CDDP and over-expression of DNMT1 reversed the pro-apoptosis effects of a miR-148b mimic [57]. DNMT1 also modulated CDDP-resistance in ovarian cancer cells by targeting miR-152 and miR-185 [59].

MiR-212 inhibits CDDP-induced apoptosis by directly targeting AChE-S in NSCLC cells [60]. Lu and colleagues examined different types of the acetylcholinesterase (AChE) and its alternative splicing forms like synaptic AChE (AChE-S), readthrough AChE (AChE-R) and erythrocyte AChE (AChE-E). They indicated that the AChE-S plays a role in CDDP-stimulated cell apoptosis. A study from Donzelli et al. suggested that mutant p53R175H (a hotspot p53 mutant) induces miR-128-2 expression, which further up-regulates the expression of p21^waf1^ by suppressing the E2F5 transcriptional repressor activity on the p21^waf1^ promoter [61]. Consecutively, over-expressed p21^waf1^ protein is translocated from nucleus to cytoplasm, where it binds to the pro-caspase-3, thereby executing its anti-apoptotic function. Altogether, transcriptional induction of miR-128-2 results in an increased CDDP-resistance of NSCLC cells.

Another miRNA which is included in CDDP-resistance development in lung cancer cells is miR-10a. Down-regulation of miR-10a induces cell apoptosis, whereas its up-regulation might inhibit apoptosis. MiR-10a suppressed MDR1, MRP1 and RhoE expression, thus enhancing the CDDP efflux and cell resistance to CDDP [62]. Xie et al. showed that upregulation of miR-218 activates cell apoptosis and promotes sensitivity of NSCLC to CDDP. They showed that miR-218 directly targets and negatively regulates the RUNX2 gene. In contrast, down-regulation of miR-218 induces chemoresistance to CDDP [63].

Up-regulated miR-26a induces cell apoptosis and increases cells sensitivity to CDDP by targeting HMGA2, whereas down-regulation of miR-26a shows the opposite effects. *In vivo* and *in vitro* experiments demonstrated that miR-96 targets SAMD9 in NSCLC cells. Up-regulated miR-96 suppressed cell apoptosis by down-regulating the SAMD9 [64]. Down-regulation of miR-141 increased the sensitivity to CDDP and influenced cell apoptosis by targeting PDCD4 [65].

One recent study suggested that miR-379 was down-regulated in NSCLC cell lines and tumor tissue [66]. Furthermore, miR-379 expression was lower in chemoresistant in comparison to chemosensitive tumors, as well as in more aggressive NSCLC cells (A549 and H1299) and their CDDP-resistant sublines. Manipulation of miR-379 expression level showed that miR-379 influences CDDP-induced apoptosis in NSCLC cells by directly targeting the eukaryotic initiation factor 4G (EIF4G), therewith acting as a tumor suppressor.

### MiRNAs and drug uptake in CDDP-resistant NSCLCs

In order to overcome toxic drug effects and modify their chemoresistance, cancer cells can deregulate the expression of different transporter-molecules responsible for drug influx and efflux. One large protein family containing active transmembrane proteins responsible for modulation of cell metabolism and cellular toxicity is the family of ATP-binding cassette (ABC) transporters. In humans, this protein family contains 48 members classified into 7 distinct subfamilies, based on their sequence and gene structure homology. There is accumulating evidence that the expression level of some of these transporters is regulated by different miRNAs.

MiR-31 was detected as a factor involved in CDDP-resistance in NSCLC. Upregulation of miR-31 induces CDDP-resistance and inhibits its direct target ABCB9, an ATP-binding cassette, sub-family B member 9. Mir-31 expression is increased in CDDP-resistant NSCLC cell lines and inversely correlated with ABCB9 expression, the inhibition of which is required for CDDP resistance development [67]. Thus, miR-31 might be used as a biomarker to predict NSCLC patients’ response to chemotherapy. Upregulation of miR106a in CDDP-resistant A549 cells (A549/CDDP) may induce CDDP-resistance by directly targeting ABCA1, a member 1 of the human transporter subfamily ABCA [68]. Seven different transporter genes able to regulate drug uptake (*ABCA1, ABCC5, ABCC6, ABCC9, ABCD2, ABCG2* and *ABCG)* have been identified by three different algorithms. ABCA1 showed the highest frequency for a putative binding site of miR-106a, which has been confirmed by an experimental approach. Overexpressed miR106a significantly decreased ABCA1, thereby reducing the CDDP uptake and CDDP-induced apoptosis in A549/CDDP cells [68]. This suggested that miR-106a might be a biomarker for prediction of a clinical response to CDDP therapy. Recently, it was shown that miR-185-5p modifies CDDP-resistance by targeting ABCC1 (ATP-binding, cassette, subfamily C member 1), a relevant tumor suppressor [69].

A study from Song et al. showed that miR - 495 is downregulated in A549/CDDP cells and CDDP - resistant tissues, compared to parental A549 cells and CDDP - sensitive tissues [70]. Significant down-regulation of miR-495 was also found in 56 NSCLC tissue samples in comparison to adjacent non-malignant lung tissue [71]. In other cell types this miRNA works as an oncogene [72], or as a tumor suppressor [73]. In NSCLC cell lines, miR-495 suppresses the copper transporting P-type adenosine triphosphatase A (ATP7A), and thereby regulates the intracellular CDDP concentration [70]. These data are supported by previous studies showing that ATP7A is involved in CDDP-resistance in various tumor cell lines like NSCLC, ovarian cancer, oral squamous cancer and epidermoid cancer cells [74–77]. As each miRNA usually has different target genes, the MTA3 (metastasis-associated protein 3), a protein frequently over-expressed in NSCLC tissue, was also identified as a target of miR-495 [71]. Experimental data show that over-expression of miR-495 inhibits proliferation, colony forming ability and migration, and leads to cell accumulation in the G0/G1 phase. Although the connection between MTA3 and CDDP-resistance was not described, these results support the role of miR-495 in CDDP-resistance. MiRNAs involved in CDDP-resistance of NSCLC cells, which are mainly assigned to drug uptake, are summarized in Table 3.

**Table 3:** MiRNAs involved in other pathways responsible for resistance of NSCLC cells to CDDP

miRNA Migr. / Inv. / Met.	Targets	Cells / Tissue	Expression	Function	Reference
miR-17 family (20a, 20b)	TGFßR2	A549/CDDP	down	TGFßR2 suppressor, reverses CDDP-resistance and suppresses metastases	[81]
miR-21	PTEN	Serum, NSCLC cells	up	Promotes migration, invasion and metastasis	[9]
mir-26b	PTEN	NSCLC tissue, NSCLC cells	down	Regulates NSCLC migration and CDDP-chemosensitivity	[78]
miR-27a	RKIP	A549/CDDP	up	Regulates EM-transition and CDDP-resistance	[82]
miR-98	PAK1	NSCLC tissue, A549/PAR	down	Inversely regulates PAK1, inhibits migration and invasion	[51]
miR-182	PDCD4	A549 cells	up	Induces resistance to CDDP by targeting PDCD4	[84]
miR-200c	n. d.	NSCLC cell lines (n=9)	down	Inversely correlates with aggressive, invasive and chemoresistant phenotype	[8]
miR-206	MET	NSCLC tissue, A549/CDDP, H1299/CDDP	down	Inhibits CDDP-resistance via MET dependent PI3K/AKT/mTOR pathways	[80]
miR-217	KRAS	NSCLC tissue, A549/PAR	down	Inhibits proliferation, migration and invasion, promotes apoptosis and sensitivity to CDDP	[79]
**DNA damage response**					
miR-17/miR-92 families	TGFßR2, CDKN1A, RAD21	A549/CDDP	down	Influence CDDP-resistance by synergistic and combined regulation of their target genes	[127]
miR-33b-3p	P21WAF1	A549/CDDP	up	Impairs the DNA damage response	[128]
miR-92					
miR-138	ERCC1	A549/CDDP	up	Negatively regulates ERCC1 expression	[38]
miR-155	APAF-1	NSCLC tissue, A549/PAR	up	Inhibits sensitivity to CDDP via negative regulation of Apaf-1	[37]
miR-509-3-5p	PLK1	CDDP-treated A549/PAR	down	Tumor suppressor, causes aberrant mitosis and anti-proliferative effects by suppressing PLK1	[20]
miR-630	ATM, H2AX, p53	A549/PAR CDDP-treated	up	Blocks DNA-damage response	[13]
**Drug uptake**					
miR-15b	PEBP4	A549/CDDP, NSCLC tissue	up	Suppresses PEBP4 expression and contribute to CDDP-resistance	[129]
miR-31-p	Dicer	A549 cells	n. d.	Enhances sensitivity to CDDP by repressing Dicer	[130]
miR-34a	PEBP4, MYCN	A549/PAR	up	Modulates CDDP-sensitivity in NSCLC cells by regulating PEBP4 expression	[131]
miR-106a	ABCA1	A549/CDDP	up	Induces CDDP-resistance by targeting ABCA1 and inhibiting apoptosis	[68]
miR-148b	DNMT1	A549/CDDP, SPC-A1/CDDP	down	Influence CDDP-sensitivity, cell viability and apoptosis	[57]
miR-181a	PTEN	A549/CDDP	up	Regulates epithelial-mesenchymal transition and metastatic properties of NSCLC cells	[46]
miR-185-5p	ABCC1	A549/CDDP	down	Promotes CDDP-induced apoptosis by suppressing ABCC1	[69]
miR-495	ATP7A	NSCLC tissue, A549/CDDP	down	Negatively regulates ATP7A and modulates CDDP-induced apoptosis	[70]
miR-503	FANCA	NSCLC tissue, A549/CDDP	down	Regulates CDDP-resistance by targeting FANCA	[102]

n. d., not defined; Expression = expression in CDDP-resistant cells or NSCLC tissue in comparison to sensitive cells or non-malignant lung tissue.

### MiRNAs and cell migration, invasion, and metastasis in CDDP-resistant NSCLCs

The development of tumor metastases is responsible for poor prognosis and for less effective treatment of cancer in general and NSCLC in particular. In the present literature, numerous correlations between different miRNAs and development of lung cancer metastases have been described. A reverse correlation between miR-200c expression and *in vitro* cell invasion, as well as *in vivo* metastasis formation has been shown [8]. In NSCLC tumor specimens (*n* = 69) decreased miR-200c expression was associated with aggressive tumor behavior, poor tumor differentiation and significantly elevated number of lymph node metastases. In addition, transfected NSCLC cells overexpressing miR-200c show an increased cytotoxicity upon CDDP treatment, which is primarily based on the finding of an elevated apoptotic rate. Based on all these data, it seems that suppressed expression of miR-200c is tightly connected with an aggressive, undifferentiated, metastatic and CDDP-resistant phenotype at least in some NSCLCs.

One important characteristic of cancer cell metastases is cell migration. MiR-26b has been shown to be downregulated in different NSCLC cells and tumor specimens [78]. Although *in vitro* up- or down-regulation of miR-26b do not have any effect on NSCLC cell proliferation, tumor cells overexpressing miR-26b show significantly increased cell migration and CDDP-resistance. It was demonstrated that PTEN acts as a direct target of miR-26b in H1299 and A549 NSCLC cell lines, and that all those effects can be reversed by PTEN overexpression [78]. Depending on the cell type, miR-217 can act either as an oncogene or as a tumor suppressor gene. MiR-217 was down-regulated in 77% of lung cancer samples (*n* = 100) compared to adjacent non-malignant tissue [79]. Overexpression of miR-217 suppresses the proliferation of lung cancer cells *in vitro* and tumor growth *in vivo*. MiR-217 also inhibits cell migration and invasion of lung cancer cells by targeting KRAS and thereby enhancing lung cancer cell sensitivity to CDDP. This strongly suggests that miR-217 works as a tumor suppressor in lung cancer.

Down-regulation of tumor suppressors or up-regulation of oncogenes facilitates cancer development. Yang et al. showed that miR-98 is down-regulated in NSCLC cells and tissue and is directly targeting PAK1, a family member of serine/threonine p21-activating kinases, which regulate cell motility and morphology [51]. MiR-98 inhibits cell proliferation, migration, invasion, and apoptosis in NSCLC cells, whereas overexpression of PTEN leads to exactly opposite effects. These data put PTEN in light as a major miRNA-regulated target responsible for CDDP-resistance of NSCLC cells. MiR-206 is one of the best characterized miRNAs and its down-regulation has been described in various cancer types. Recently, in CDDP-resistant NSCLC cell lines down-regulation of miR-206 was associated with low CDDP sensitivity [80]. *In vitro* and *in vivo* overexpression of miR-206 reduced resistance, migration and invasion of CDDP-resistant cells and suppressed EMT by directly targeting the proto-oncogene MET and its downstream PI3K/AKT/mTOR pathway. Taken together, these data indicate that overexpression of miR-206 and/or inactivation of its target genes might reverse the CDDP-resistance in lung adenocarcinoma cells, leading to a better response to CDDP therapy.

Some members of the miRNA-17 family (miR-17, miR-20a and miR-20b) have a potential as key regulatory factors in CDDP-resistance. Down-regulation of all three members were detected in CDDP-resistant A549 cells in comparison to sensitive cells. The TGF-ß receptor 2 (TGFßR2) was detected as a direct target of these three miRNAs [81]. Overexpression of miRNA-17, 20a and 20b decreases both CDDP-resistance and EMT, and increases migration of CDDP-resistant A549 cells. Altogether, this indicates that miRNA-17 family members at least partially influence CDDP-resistance and participate in metastatic mechanisms of NSCLC cells. Further studies would be necessary to define whether miR-17 family members are useful as predictive biomarkers for sensitivity to CDDP-based chemotherapy in NSCLC.

In comparison to parental cells, CDDP-resistant A549 cells show a prominent up-regulation of miR-27a [82]. *In vitro* inhibition of miR-27a suppressed the invasion of CDDP-resistant cells and enhanced their sensitivity to CDDP. *In vivo* experiments in nude mice, injected with A549 cells stably expressing miR-27a and treated with CDDP, show dramatic increase of lung metastasis in the miR-27a over-expressing group, with exactly opposite results in animals injected with miR-27a antagomir-transduced cells. These data indicate that miR-27a induces mesenchymal features, promotes tumor metastasis, and CDDP-resistance in NSCLC cells, by directly targeting RKIP (Raf Kinase Inhibitory Protein) [82].

Overexpression of miR-182 was found in different human cancers, including NSCLC. Mir-182 overexpression was also shown in CDDP-resistant A549 in comparison to parental cells [83]. Suppressing miR-182 expression by a specific inhibitor enhanced sensitivity of A549 cells to CDDP by negatively regulating the tumor suppressor PDCD4 (Programed Cell Death 4 Protein) [84]. As already mentioned in the context of apoptosis regulation and drug uptake, miR-181 directly targets PTEN [46]. Thereby miRNA-181 not only influences apoptosis, but also promotes invasion and migration of NSCLC cells. MiRNAs involved in CDDP-resistance of NSCLC cells, which are mainly assigned to migration, invasion and metastasis, are summarized in Table 3.

Based on the present literature, one can easily conclude that miRNAs are frequently multiplayers involved in the regulation of various target genes and cellular processes. In fact, it seems that the vast majority of miRNAs have this characteristic, which makes every classification, prediction, and understanding of possible cross-reactions very challenging.

### MiRNAs and hypoxia in NSCLCs

Hypoxia is a common microenvironmental feature and a distinctive property of solid tumors, resulting from shortness of supply with oxygen. Different cellular responses to hypoxia are frequently mediated by changes in targeted gene expression. One of the most important and well described factors deregulated under hypoxic conditions is the hypoxia-inducible factor-1 (HIF-1). Members of the HIF-family (HIF-1α, HIF-1ß and HIF-2α) bind to hypoxia-response elements (HRE) in the promoter regions of target genes, thereby acting as transcription factors and regulators of gene expression. The present literature data differ very much concerning the number of target genes, speculating that 150 to 500 genes might be regulated by HIF proteins. These target genes are involved in different cellular processes such as cell metabolism, apoptosis, proliferation and cell cycle, autophagy, angiogenesis, etc. HIF-1 consists of two subunits: hypoxia-inducible HIF-1α and non-oxygen-responsive, constitutively expressed HIF-1ß. HIF-1α is overexpressed and its overexpression is associated with increased patient mortality in different human cancers. Overexpression of HIF-1α, both at the RNA and at the protein level, was also shown in NSCLC patients and was associated with a poor prognosis. Expression of HIF proteins in lung carcinoma can also be regulated by different miRNAs. Many miRNAs have been found to be involved in the regulation of the cellular response to hypoxia, both in tumor and in non-malignant cells (for summary see Table 4).

**Table 4:** Hypoxia-deregulated miRNAs in NSCLCs

miRNA	Target genes	Correlationwith HIF-1α	Entity (regulation under HOX)	Main function of miRNA	Reference
miR-15a	n. d.	neg.	NSCLC (down)	n. d., miR-15a expression negatively correlates with HIF-1α	[132]
miR17-92 cl.	HIF-1α	neg.	Lung cancer (up)	Mediate HIF-1α inhibition of cell growth in normoxic conditions	[133]
miR-21	HIF-1α	pos.	Adenocarcinoma and radioresistant NSCLC (up)	Influences cancer cell adaptation to tumor-specific environment, promotes glycolysis and modulates radioresistance by upregulation of HIF-1α	[134–136]
miR-23a	PHD1, PHD2, ZO-1	pos.	Adenocarcinoma (up)	Enhances neovascularization and tumor growth	[137]
miR-101	Cox2, Lin28B, let-7 miRNA	neg.	NSCLC (down)	Tumor suppressive function in NSCLC	[138]
miR-143	IGF-IR, IRS1	neg.	Cr (VI)-transformed lung epithelial cells (down)	Antiangiogenic effects	[139]
miR-155	FOXO3A, TP53INP1	pos.	NSCLC (up)	Shows radio-protective effect in NSCLC cell lines	[140]
miR-191	NF1A	pos.	NSCLC (up)	Promotes cell proliferation and migration under HOX	[141]
miR-199a	HIF-1α	neg.	NSCLC (down)	Suppresses hypoxia-induced proliferation of NSCLC cell	[96]
miR-210	NDUFA4, SDHD, PTPN1, TP53I11, HOXA1, HIF-1α	pos.	NSCLC, NSCLC - late stage (up)	Influences cell viability, activates caspase 3/7, promotes double-strand break repair, and increases radioresistance in NSCLC cells.	[92, 142, 143]
miR-301b	Bim	pos.	NSCLC specimens and cell lines (up)	Enhances cell proliferation, reduces apoptosis, increases resistance to chemotherapy	[144]
miR-494	PTEN	pos.	NSCLC cells (up)	Enhances endothelial cell migration, promotes angiogenesis and tumor growth under HOX	[97]
miR-519c	HIF-1α	neg.	NSCLC cells (n. d.)	Suppresses tumor angiogenesis, growth, and metastasis	[145]
miR-622	HIF-1α	neg.	NSCLC cells (down)	Inhibits cancer cell migration and invasion	[99]
miR-1908	AKT1S1	n. d.	NSCLC (down)	Suppresses cell proliferation	[146]

HOX, hypoxia; neg., negative; pos., positive; cl., cluster; n. d., non-determined; NSCLC, non-small cell lung carcinoma; CTL, cytotoxic T lymphocytes.

One of the first, and in the meantime probably the best described miRNAs, identified in the subset of hypoxia-regulated miRNAs, is miR-210. MiR-210 has been described as a “micro-manager of the hypoxia pathway” [85] and its overexpression has been shown in numerous tumors including NSCLC [86–90]. It was shown that miR-210 expression is progressively increased with growth and invasion of tumor cells, being the highest in late-stage NSCLCs [91]. Furthermore, miR-210 expression is also correlated with a hypoxic signature in NSCLCs and is increased in A549 cells under hypoxic conditions. MiR-210 expression alters cell viability and enhances caspase-3/7 activity in A549 lung adenocarcinoma cells and increases HIF-1α activity. The authors suggest miR-210 to directly target the SDHD (subunit D of succinate dehydrogenase complex), thus revealing a positive auto-regulatory loop associating miR-210 and HIF-1α. In their subsequent study, Grosso et al. developed a NSCLC-derived cell line (A549) stably expressing miR-210, and showed a significant stabilization of HIF-1α in those cells and increased radioresistance under hypoxic conditions [92]. This is, at least partially, based on more efficient double-strand break (DSB) repair in miR-210 overexpressing cells, as shown with immunofluorescence of p53-binding protein (53BP1) and γH2AX, two markers of DSB-induced DNA repair. These findings might be highly relevant, because it is well known that hypoxic cells are more resistant to radio- and chemotherapy than normoxic cells. Although the molecular mechanism is not fully understood, it seems that miR-210 influences mitochondrial function, since overexpressing cells show enlarged mitochondria with rearranged cristae. In addition, miR-210 overexpressing cells show an increased glycolytic rate in comparison to control cells with low miR-210 expression. Based on those results, miR-210 seems to be a possible prognostic and predictive biomarker for NSCLC. This prognostic impact was further supported by a large-scale study from Eilertsen et al. showing miR-210 expression on tissue micro array data of 335 patients with stage I-IIIA NSCLC [93]. They showed that high expression of miR-210 in both cancer and stromal cells of the tumor is associated with improved survival. Concerning correlation between miR-210 and different hypoxia markers in this patient cohort, there was no correlation with carbo anhydrase IX (CAIX), but there was a significant positive correlation with both HIF1 and HIF2. The authors concluded that miR-210 might serve as a prognostic biomarker in NSCLC patients and its reintroduction may be an interesting future treatment approach. Interestingly, in their cohort with 80 NSCLC patients, Osugi et al. showed correlation of miR-210 expression with lymph node metastasis, late disease stage and poor prognosis in patients with lung adenocarcinoma [94]. Although data from those two studies are contradictory, they both suggest that miR-210 could be used as a prognostic marker in NSCLC patients.

MiR-199a suppresses the hypoxia-induced proliferation of NSCLC by targeting HIF-1α [95]. Ding et al. showed that miR-199a expression is negatively correlated with HIF-1α expression in NSCLC patients. The expression of miR-199a was significantly lower in lung tumors as compared to adjacent non-malignant tissue and was strongly negatively correlated with tumor progression. Results based on the overexpression of miR-199a in low miR-199a-expressing NSCLC cells, and on inhibition with a specific HIF-1α inhibitor PX-478 suggest that miR-199a has an inhibitory effect on the hypoxia-induced proliferation of NSCLC cells, through negative regulation of HIF-1α and blocking of HIF-1α-mediated glycolysis [96]. Altogether, these data suggested opposite roles of miR-199a and HIF-1α in NSCLC progression.

Hypoxia is well known as a primary factor stimulating angiogenesis. It has been shown that miR-494 expression in A549 cells is induced in response to hypoxia, thereby promoting cell migration, angiogenesis and tumor growth under hypoxic conditions [97]. MiR-494 is a tumor-derived pro-angiogenic stimulus upregulated via HIF-1α-mediated transcriptional activation. It also inhibits tumor angiogenesis *in vivo*, and therefore might be a promising anti-angiogenic strategy for cancer therapy. Furthermore, miR-494 expression in NSCLC tissue is higher than in non-malignant lung tissue, and was positively correlated with pathological staging of NSCLC patients. The negative correlation of increased miR-494 with the survival indicates that miR-494 is an independent prognostic factor for NSCLCs. Interestingly, in gastric cancer, miR-494 was also described as a tumor suppressor targeting c-myc [98].

Another interesting and promising miRNA responsible for metastatic spread of lung cancer cells is miR-622. Cheng and coworkers showed in A549 lung cancer cells that miR-622 directly binds to the 3´-untranslated region of HIF-1α mRNA and decreases its expression [99]. Overexpression of miR-622 efficiently reduces the HIF-1α level, thereby diminishing the migration and invasion abilities of cancer cells. Mir-622 influences and suppresses metastasis in xenograft model of lung cancer via inhibition of HIF-1α-related EMT signaling. Furthermore, miR-622 expression is regulated by the transcription factor FOXO3a. Altogether, the authors suggest that miR-622 could be a promising target for the development of miRNA-based therapeutics for metastatic lung cancer. Unfortunately, there comes another complexity into play. Not only HIF-1α expression is controlled by different miRNAs, but HIF-1α itself controls the expression of various miRNAs. However, a detailed description of those cross-links would be out of the scope of this review.

### Prognostic impact of circulating microRNAs in CDDP-resistant NSCLCs

Prognosis for NSCLC patients is associated with TNM stage and with the development of chemoresistance. Another relevant prognostic aspect for cancer patients is metastasis formation. Unfortunately, most NSCLC patients are diagnosed at advanced stages and often metastasis is already present. MiRNAs could serve as prognostic indicators in lung cancer, as shown above. It has been observed that miRNAs are closely related to tumor development and stably exist in serum/plasma. MiRNAs are resistant to RNaseA digestion and other harsh conditions, partially explaining their stability in serum.

The relationship between miR-210 expression and disease-free and overall survival of NSCLC patients was analyzed by Osugi et al. Although no significant association between MiR-210 expression and different clinical characteristics (sex, age, tumor size, lymph node status, TNM stage) was observed, miR-210 expression was significantly correlated with disease relapse. Upregulated miR-210 was associated with poor prognosis, shorter disease-free, and overall survival of patients with lung adenocarcinoma [94]. Based on correlation between miR-210 level and patient survival, it was concluded that miR-210 acts as an independent prognostic factor in lung adenocarcinoma patients. Furthermore, a significant correlation between miR-210 level in serum and disease stage in NSCLC patients was observed. The serum level of miR-210 in the control group was significantly lower than in NSCLC patients. The expression of miR-210 in NSCLC patients was significantly correlated with higher clinical stage and with the presence of lymph node metastasis. MiR-210 level was also higher in patients with stable disease in comparison to patients with partial response to CDDP therapy [100]. This suggests that miR-210 is a good candidate as a diagnostic and prognostic biomarker in NSCLC.

MiR-216a was down-regulated in NSCLC specimens in comparison to non-malignant lung tissue [12]. Decreased expression was inversely correlated with cancer stage, metastasis, and poor survival of NSCLC patients. Down-regulation of miR-216a was associated with increased NSCLC cell growth and metastasis. Furthermore, decreased miR-216a suppressed CDDP-induced apoptosis and cell growth inhibition in NSCLC cells, thus increasing the resistance to CDDP. Therefore, in NSCLCs miR-216a may have a role as cancer suppressor and might act as biomarker for predicting NSCLC progression.

As already mentioned in this paper, miR-503 modifies NSCLC cell resistance to CDDP by influencing expression of different target genes like Bcl-2, FANCA, PI3K p85, and IKK-β [55, 56, 101]. Compared to non-malignant lung tissue, in NSCLC tissues, significant down-regulation of miR-503 has been detected and miR-503 levels were associated with advanced tumor stage and poor prognosis [102]. Kaplan-Meier analyses showed a worse survival of patients with lower miR-503 expression as compared to NSCLC patients with a higher expression. Based on this data and further multivariate analysis, the authors suggested that miR-503 might be an independent prognostic biomarker for overall survival in NSCLC patients.

MiR-638 was also an independent prognostic factor in NSCLC patients [11]. Interestingly, miR-638 expression in NSCLC cells is induced by CDDP-treatment both *in vitro* and *in vivo*. Despite all those promising results, it must be stressed that the exact molecular mechanisms of miR-638 in lung cancer tumorigenesis are not well known.

Circulating miR-125b was analyzed in advanced inoperable NSCLC patients who received CDDP-therapy [103]. High levels of circulating miR-125b were significantly positively correlated with cancer stage and with tumor differentiation status and poor prognosis after adjuvant chemotherapy in NSCLC patients. Thus, miR-125b might represent a biomarker for chemotherapeutic response and prognosis for NSCLC patients.

MiR-21 expression studies in plasma of NSCLC patients, in comparison to age- and sex-matched healthy controls, show significantly increased miR-21 levels [52]. Furthermore, the miR-21 level is correlated with TNM stage and with response to CDDP chemotherapy. Correspondingly, the authors suggest to use miR-21 as a biomarker for early diagnosis and as a predictive marker for response to platinum-based chemotherapy.

The expression of miR-107 in patients at different TNM stages was analyzed by Zhong et al. [104]. The level of miR-107 in patients with TNM stage II and IIIa was lower in comparison to patients with TNM stage I. The expression level of miR-107 was also decreased in NSCLC tissue samples compared to adjacent non-malignant lung tissue. Mechanistically, it was shown that miR-107 directly targets cyclin dependent kinase 8 (CDK8), thus making NSCLC cells more sensitive to CDDP-therapy [21]. Thus, a low level of miR-107 is correlated with increased lung cancer progression and limited survival in NSCLC patients, suggesting that miR-107 might be used as a prognostic biomarker and to predict the response to CDDP-therapy in NSCLC patients.

Gallardo et al. showed that decreased levels of miR-34a in NSCLC patients who underwent curative surgery were correlated with an increased rate of relapse. In patients with reduced miR-34a expression, frequent mutations of the tumor suppressor p53 were observed. Patients with both decreased miR-34a levels and p53 mutations had a particularly poor prognosis, indicating that presence of these two factors leads to shortened survival of NSCLC patients after curative surgery [105]. Therefore, also miR-34a was suggested as a prognostic marker in NSCLC patients.

## CONCLUSIONS

NSCLC has a very high mortality rate as compared to other cancers, and NSCLC patients are more likely to survive if they are diagnosed early and received curative therapy. Good biomarkers, predicting the response to CDDP, might help clinicians to make timely therapy decisions and maybe even to improve survival. Over the past few years, at lot of evidence has accumulated to suggest aberrant expression of miRNAs in cancer patients as compared to healthy controls. MiRNA expression may be strongly correlated with cancer development, survival, and CDDP resistance. MiRNAs are released from primary tumor cells into the peripheral circulation, where they circulate in a stable level, due to resistance to RNase digestion and other elimination factors. The high stability of serum miRNAs makes them especially interesting and promising as potential noninvasive biomarkers for NSCLC detection and as prognosticators. In addition, miRNAs and miRNA-inhibitors have a potential to be an adjunct to lung cancer treatment. In conclusion, better understanding of the molecular mechanisms behind particular miRNAs and of their role in tumor initiation, growth and development of metastases might improve diagnosis and prognosis of NSCLC patients.

## Abbreviations

NSCLC: non-small cell lung carcinoma
ADC: adenocarcinoma
SCC: squamous-cell carcinoma
LCC: large-cell carcinoma
CDDP: cis-diaminedichloroplatinum (cisplatin)
UTR: untranslated region
miRNA: micro RNA
EMT: epithelial-to-mesenchymal transition
GSH: glutathione
GSTP1: glutathione s-transferase P1
eIF4B: eukaryotic initiation factor 4B
ZEB1: zinc finger and homeobox transcription factor-1
PTEN: phosphatase and tensin homolog
SMAD7: SMAD family member 7
p27Kip1 (CDKN1B): Cyclin-dependent kinase inhibitor 1B
CDK: cyclin-dependent protein kinase
PLK1: polo-like kinase 1
CDC: cell division cycle related protein kinase
DDIT4: DNA damage inducible transcript 4
PARP3: poly(ADP-ribose) polymerase family member 3
EP300: E1A binding protein P300
Bcl-2: apoptosis regulator Bcl-2
VEGF-A: vascular endothelial growth factor A
YAP1: yes-associated protein 1
CCNE1: cyclin E1
HDGF: hepatoma-derived growth factor
Bcl-XL: B-cell lymphoma – extra large
Apaf-1: apoptotic peptidase activating factor 1
ERCC1: DNA excision repair cross-complementation group protein 1
EZH2: enhancer of zeste homolog 2
CCND3: cyclin D3
PDK1: 3-phosphoinositide-dependent protein kinase 1
FOXP4: forkhead box P4
GIT1: G-protein-coupled receptor kinase-interacting protein 1
SEMA4C: semaphoring 4C
LIMK1: LIM domain kinase
BAX: Bcl-2 associated X
EMT: epithelial-mesenchymal transition
HMGA2: high mobility group A2
PAK1: p21-activated protein kinase
NF-κB: nuclear factor kappa B subunit 1
FANCA: Fanconi anemia complementation group A protein
DNMT1: DNA (cytosine-5)-methyltransferase 1
AChE: acetylcholinesterase
MDR (ABCB1): ATP binding cassette subfamily B member 1
MRP1 (ABCC1): ATP binding cassette subfamily C member 1
RUNX2: runt related transcription factor 2
SAMD9: sterile alpha motif domain containing 9
PDCD4: programmed cell death 4
ATP7A: ATPase copper transporting alpha
MTA3: metastasis-associated protein 3
mTOR: mechanistic target of rapamycin
TGFßR2: transforming growth factor beta receptor 2
RKIP: raf kinase inhibitory protein
Hif-1: hypoxia-inducible factor
53BP1: p53-binding protein 1
CAIX: carbonic anhydrase 9
FOXO: forkhead box
